# An Efficient Automatic Gait Anomaly Detection Method Based on Semisupervised Clustering

**DOI:** 10.1155/2021/8840156

**Published:** 2021-02-15

**Authors:** Zhenlun Yang

**Affiliations:** School of Information Engineering, Guangzhou Panyu Polytechnic, Guangzhou 511483, China

## Abstract

The aim of this work is to develop a common automatic computer method to distinguish human individuals with abnormal gait patterns from those with normal gait patterns. As long as the silhouette gait images of the subjects are obtainable, the proposed method is capable of providing online anomaly gait detection result without additional work on analyzing the gait features of the target subjects before ahead. Moreover, the proposed method does not need any parameter settings by users and can start producing detection results under the work by only collecting a very small number of gait samples, even though none of those gait samples are abnormal. Therefore, the proposed method can provide fast and simple deployment for various anomaly gait detection application scenarios. The proposed method is composed of two main modules: (1) feature extraction from gait images and (2) anomaly detection via binary classification. In the first module, a new representation of the most frequently involved area of the silhouette gait images called full gait energy image (F-GEI) is proposed. Furthermore, based on the F-GEI, a novel and simple method characterizing individual walking properties is developed to extract gait features from individual subjects. In the second module, based on the very limited prior knowledge on the target dataset, a semisupervised clustering algorithm is proposed to perform the binary classification for detecting the gait anomaly of each subject. The performance of the proposed gait anomaly detection method was evaluated on the human gaits dataset in comparison with three state-of-the-art methods. The experiment results show that the proposed method is an effective and efficient gait anomaly detection method in terms of accuracy, robustness, and computational efficiency.

## 1. Introduction

In recent days, more and more automated gait anomaly detection systems need to be deployed and used to generate instant online results in various application scenarios, such as the potential disease alert in a nursing home for the elderly and alcohol usage alert in the parking lots. As defined in the literature, in online anomaly gait detection, the anomaly is identified by performing the predictive analysis in real time with each new observation [1]. On the one hand, in such application scenarios, the anomaly detection systems need to be used with fast deployment without collecting many gait samples or analyzing the features of the specified subjects. In such application scenarios, the labeled samples of at least one individual subject with abnormal gait is hard to be collected before ahead, and even the unlabeled data of individuals are not sufficient for the classifier training before the gait analysis system starts to work. On the other hand, prior knowledge about the studied walking subject is also hard to be acquired. Moreover, the detected target subjects in different scenarios have various features in both gait postures and behavior patterns. However, according to existing research studies, the solution to these applications is based on analyzing the scenarios individually and independently. Each application scenario requires the participation of professional researchers and technical engineers, and there is a relatively long period between demand and actual system deployment. If the needs of an application are not extensive, it may be difficult to get enough investment and attention. Therefore, the development of a single common gait anomaly detection solution for these various applications is necessary.

For various application scenarios, this study aims at developing a common automatic anomaly gait detection method providing fast and simple deployment supported by the three advantages: (1) do not need additional work on analyzing the target subjects' gait features before ahead, (2) do not need parameter tuning by users, and (3) only need to collect a tiny number of labeled samples before running.

Without any requirement for additional work before ahead, the gait anomaly detection system based on the proposed method can be deployed on the target spots directly. And after the first few individuals are labeled by visual inspection performed by experienced persons, the gait anomaly detection system based on the proposed method starts working to generate the real-time result of whether the current individual subject has an abnormal gait when the subject is passing the detecting point.

The proposed anomaly gait detection method, abbreviated as AGD-SSC, which stands for Anomaly Gait Detection based on Semisupervised Clustering, is composed of feature extraction from gait images and anomaly detection via binary classification. And, there are three key strengths of the proposed AGD-SSC method to provide the application potentiality in many scenarios:

1. The gait collection for the target subjects is based on the depth imaging technology, which has good invariance against illumination changes and significantly simplifies background subtraction and segmentation. The gait pattern is generated by a newly proposed simple incremental method based on gait energy image (GEI), and the method is less sensitive to noise. Therefore, the proposed AGD-SSC method has good environmental adaptability and a wide range of application prospects.
2. A features' extraction method based on the deviation from the normal gait pattern is proposed and characterized by not depending on the complete gait cycle identification and the gait frames' synchronization for each detected individual subject. This greatly reduces the difficulty and complexity of the gait image processing and improves the overall speed and accuracy of the proposed method.
3. A semisupervised clustering algorithm that requires only a small number of labeled samples of the dataset is proposed to provide fast and accurate classification results. Moreover, the clustering algorithm is with implicit parameters and does not need any parameter settings by users.

The rest of the paper is structured as follows. Related previous work on automatic gait anomaly detection is reviewed in Section 2.  Section 3 briefly introduces the two main existing methods which provide the foundations for the proposed method.  Section 4 presents the proposed AGD-SSC method.  Section 5 discusses the experimental results. Finally, Section 6 concludes the study and gives further research recommendations.

## 2. Related Work

Gait is a basic and well-trained behavior that contains information about the identity and state (e.g., actions, intentions, and emotions) of an individual subject [2]. The gait of an individual contains a wealth of information. It can be recorded as images or videos easily in a noncontact process without the cooperation of the detected individual. The gait anomalies are the important symptoms of some potential problems such as brain disorders or lameness of the individuals. Moreover, the application systems based on the automatic gait analysis are quicker and more cost-effective than traditional procedures. Therefore, in recent years, automatic gait anomaly detection for the subjects of human or large farming animals has attracted much interest in terms of its application in

1. The early detection of potential health problems for the elderly or patients living at home [3]
2. The diagnosis of brain disorders [4] and the prognosis of surgery [5]
3. The alcohol usage detection for the people [6]
4. The anomaly detection of the body condition, such as lameness, of large farming animals (cattle, pigs, and sheep) [7–9]

Information about gait encompasses a variety of patterns, including head bob, leg swing, and trackways. The automatic gait anomalies can be described by various methods and assessed with various metrics. Therefore, the term gait anomalies' analysis embraces an extensive and diversified literature. From the viewpoints of analytical objectives, there are two main approaches. Some studies have challenged to obtain the type of anomaly from multiple anomalies classes [10–12]. Some other research studies focused on performing the binary classification task to identify abnormal gait patterns of the subjects [13–16]. The binary anomaly gait detection methods only need to distinguish the individuals with unusual postures or movements and which means lower requirements on the input data and easy system implementation. Therefore, the binary anomaly gait detection methods have become a focus of recent research studies.

Various techniques have been proposed for representing and analyzing gait data to achieve anomaly gait detection. According to the literature, two main approaches, the nonmachine learning-based approach and the machine learning-based approach, are used to provide the anomaly detection results [17]. For their capabilities of online detection with limited input data for anomaly gait classification, the nonmachine learning-based approaches such as statistical analysis-based techniques [18, 19] and mathematical transforms-based techniques [20, 21] have been applied to various applications. Nevertheless, the nonmachine learning-based approaches have two vital drawbacks: (1) the prior knowledge about the studied walking agent must be acquired before the analysis; (2) since the adopted body parameters of different agents are various in different applications, a specific method based on the nonmachine learning technique only can be used in a single specified application scenario. The machine learning-based approaches have attracted much attention from the research community for their abilities on both capturing patterns and modeling complex nonlinear relationships in gait data [22]. It should be noticed that there are some existing machine learning-based common anomaly detection algorithms such as Artificial Neural Network (ANN) [23], Bayesian Network (BN) [24], Minimum Covariance Determinant Estimator (MCD) [25], One Class Support Vector Machine (1-SVM) [26], Local Outlier Factor (LOF) [27], Isolation Forest (IF) [28], and Cluster-based method [29]. However, whether these algorithms can be applied to the gait anomaly detection depends on the characteristics of the corresponding feature vectors from gait sequences and the prior knowledge about the dataset.

A big part of the existing machine learning-based gait anomaly detection approaches use the supervised machine learning methods [22], such as Artificial Neural Network (ANN) [30, 31] and Support Vector Machine (SVM) [13, 32, 33]. These techniques have been investigated extensively for the automatic recognition of gait patterns. The main drawback of the supervised machine learning methods is the rigid need for training data with labels. Generally, the jobs of collecting training data and labeling are both time and resource consuming. And, such a data-intensive requirement can be a huge barrier to the implementation of the methods. In some cases, labels can be extremely difficult or even impossible to obtain. As a result, the target applications of the automatic gait analysis systems based on supervised machine learning methods are limited.

If very little prior knowledge about the dataset is known, unsupervised machine learning methods can be used to detect the gait anomaly, such as the *K*-means clustering method for lameness classification of cows [10] and the 1-SVM and IF methods for gait disorder detection of human [34]. However, the gait anomaly detection result is affected by some key parameters of the algorithms, and these parameters are hard to tune soundly without enough prior knowledge on the application domain or the dataset. Moreover, the approaches based on unsupervised machine learning suffer from time-consuming problems. Therefore, the amount of unsupervised machine learning methods in this context proposed in recent years is relatively small.

Semisupervised learning approaches that exploit both unlabeled and labeled samples are capable of avoiding the problems of demanding large amounts of training data with labels in the supervised learning approaches and the environment and parameters' dependency problems in the unsupervised learning approaches. Yang et al. [35] developed a method that can automatically detect near-miss falls based upon a worker's kinematic data and a semisupervised classifier. Mikos [36] presented a FOG (freezing of gait) detection method for Parkinson's disease patients with semisupervised neural networks. Besides, the literature on gait anomaly detection, some gait classification methods are also based on semisupervised learning algorithms. Such as Li et al. [37] had proposed two semisupervised gait classifiers based on self-training on both few labeled data samples and large number of unlabeled data samples. However, those existing semisupervised learning approaches suffer from at least one of the drawbacks as follows: (1) at least one labeled sample from each target class are required before they can be used to classify the new individuals, (2) the number of unlabeled data samples needed to train the classifier is large, and (3) the detection accuracy is relatively low. Therefore, the existing semisupervised learning approaches are nevertheless impractical in some real-world gait anomaly detection applications.

## 3. Background for the Proposed AGD-SSC

In this section, the two essential ideas adopted by the proposed AGD-SSC method are introduced. In Section 3.1, the concept of the gait energy image (GEI) for the feature extraction module in the proposed method is given. And, a semisupervised clustering method called COP-*K*-means for the classification module in the proposed method is discussed in Section 3.2.

### 3.1. Gait Energy Image (GEI)

Gait presentation is vital in gait classification. Various gait presentation techniques have been proposed in the past decades, and these methods can be divided as model-based and model-free approaches. For model-based approaches, a predefined model is required by taking advantage of the prior knowledge from the shape and dynamics of individual bodies. However, prior knowledge on the target agent is difficult to obtain in many real-world applications. Therefore, the model-based gait presentation approaches can only be used in some specified target applications. On the contrary, the model-free approaches can be used in many applications without prior knowledge of the subjects' bodies. Moreover, the model-free approaches are less sensitive to the sharpness of the target subjects. Therefore, model-free approaches have attracted increased attention from researchers in recent years.

One of the most common model-free approaches is Gait Energy Image (GEI) which is a spatiotemporal gait representation using gray-level average silhouettes from videos or across images of a gait cycle [38]. The canonical gait representations based on silhouettes treat gait as a sequence of templates. On the contrary, gait is represented in GEI with a single image that contains information about both major body shapes of silhouette contours and their changes over the gait cycle to characterize human walking properties. Given an agent walking sequence, the normal GEI generation procedure can be summarized that (1) the silhouette is extracted from each frame using some specified methods, (2) the preprocessing is performed on the silhouette image sequence, which includes size normalization and horizontal alignment on each silhouette image in the gait sequence, (3) the silhouette images of the complete gait cycle(s) overlap together and generate the GEI with the equation as follows:

(1) $$\begin{array}{c} G\left(x,y\right)=\frac{1}{N}\overset{N}{\underset{t=1}{\sum }}B_{t}\left(x,y\right), \end{array}$$

where *B*_*t*_(*x*, *y*) is the gray value of binary gait silhouette image in the coordinate (*x*, *y*) at *t* in a sequence and *N* is the number of gait silhouette images in the complete cycle(s) of a sequence.

From the equation above, it can be seen that each silhouette image is representing the space-normalized energy of the subject walking at the corresponding moment and GEI is presenting the time-normalized accumulative energy of the subject walking in the complete cycle(s). If a pixel in GEI is with higher gray value, it can be said that the duration of the foreground staying at this position is long and the position of the pixel is with higher energy.

GEI can represent motion in a more compact way. When the noise at different moments is uncorrelated and identically distributed, GEI is less sensitive to silhouette noise in individual frames such as holes, shadows, and missing parts. It has been demonstrated that GEI has competitive performance against some alternative representations in gait recognition. Although some improved varies based on GEI have been proposed in recent years, such as gait history image (GHI) [39], gait moment image (GMI) [40], frame difference energy image (FDEI) [41], and skeleton gait energy image (SGEI) [42], GEI is still the most efficient approach with unambiguous and uncomplicated operation steps.

Considering the need to extract features from various target objects in various applications, GEI is adopted in the proposed AGD-SSC method.

### 3.2. COP-*K*-Means Algorithm

For the problem to classify a given dataset *X* into *K* nonintersection clusters, the *K*-means clustering algorithm is one of the most frequently employed methods [43] when no prior knowledge about the dataset is known. In the *K*-means clustering algorithm, the number of clusters *K* is assumed to be fixed, and if an instance *x*_*i*_ in *X* is more similar to the center of the cluster *C*_*j*_ of the *K* clusters than to the cluster centers of rest clusters, *x*_*i*_ will be assigned to a cluster *C*_*j*_.

However, in real-world applications, although there is not much prior knowledge about the domain or the dataset, the prior knowledge does exist and could be very useful in clustering the data. The prior knowledge could be the general knowledge about the target application domains. For example, in the application scenarios of the detection of alcohol usage for people in the parking lots, the assumption that most of the individuals are normal and only a small proportion of the individuals are with abnormal gait is satisfied. Besides, some other prior knowledge about the dataset is the labels of some of the individuals involved in clustering which are known in advance.

Apparently, the direct *K*-means clustering algorithm has no way to take advantage of the prior knowledge. Therefore, some modified *K*-means algorithms were proposed to make use of prior knowledge to perform the semisupervised clustering. Semisupervised clustering algorithms aim to improve clustering results using limited supervision. Wagstaff et al. [44] incorporated prior knowledge in the form of instance-level constraints to the clustering problem and proposed a semisupervised clustering method, named the COP-*K*-means algorithm, to enforce the constraints to be satisfied. The COP-*K*-means algorithm takes in a dataset *X*, a set of must-link constraints specify that two instances must be placed in the same cluster, a set of cannot-link constraints specify that two instances must not be in the same cluster, and it returns a partition of the instances in *X* that satisfies all specified constraints. Since then, as a most common form of semisupervised clustering, some other constrained *K*-means clustering variants [45–47] were also developed and received a significant amount of attention in the machine learning and data mining communities.

The pseudocode of the COP-*K*-means is given in Algorithm 1. And, more details of the COP-*K*-means can be found in [44].

**Figure 1:**
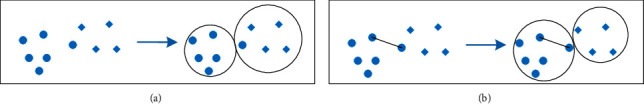
A demonstration of the COP-*K*-means clustering.

The advantage of the COP-K-means against the direct K-means is demonstrated in Figure 1, which shows a binary classification task on a dataset consisting of two classes of data points. All the circular data points in Figure 1 belong to one class, while all the diamond data points belong to the other class.  Figure 1(a) shows the classification result by direct *K*-means. It is clear that one circular data point closest to the boundary of the diamond data points is misclassified to the class of diamond data points.  Figure 1(b) shows the classification result by COP-K-means. Different from Figure 1(a), there is a link between the circular data point, which is misclassified Figure 1(a), and another circular data point. It is a must-link constraint, a form of the prior information about the dataset, which specifies that the two data points have to be in the same cluster. Supported with the must-link constraint, the classification result by COP-K-means is more accurate.

## 4. The Proposed AGD-SSC Method

**Figure 2:**
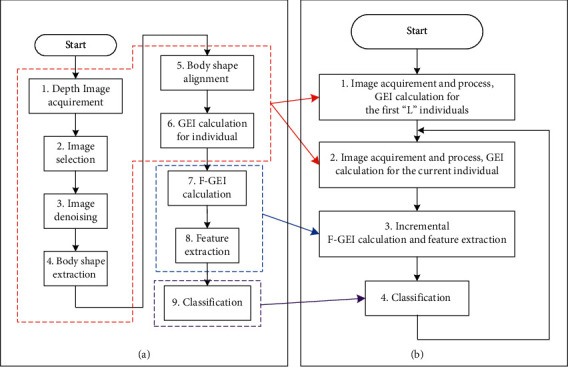
The flowchart of the proposed AGD-SSC method.

The proposed method is composed of two main modules: (1) feature extraction from gait images and (2) anomaly detection via binary classification. Nevertheless, some preliminary works, such as image capturing, image selecting, and image denoising, need to be carried out before feature extraction from gait images. Therefore, in order to describe the proposed AGD-SSC method in a more clear and precise manner, we divide the whole processing flow of the AGD-SSC into 9 sequential operations, and the corresponding flowchart is shown in Figure 2(a). The 1^st^ operation to the 8^th^ operation are related to the feature extraction module, while the 9^th^ operation is related to the binary classification module.

The flowchart in Figure 2(a) presents every single operation of the proposed AGD-SSC method. However, the target application of the proposed AGD-SSC method is the online anomaly gait detection for the walking subjects passing through the detection point individually. For such an online anomaly gait detection application scenario, the proposed AGD-SSC method includes 4 steps given in Figure 2(b). Each of the 4 steps includes one or more operations of the flowchart in Figure 2(a). The 1^st^ and the 2^nd^ steps in Figure 2 (b)are composed of the first 6 operations in Figure 2(a), while the 3^rd^ step is composed of the 7^th^ and the 8^th^ operations in Figure 2(a). And, the 4^th^ step in Figure 2(b) corresponds to the 9^th^ operation of Figure 2(a).

Without any prior knowledge, it is impossible to tell whether the gait of an individual is abnormal or not. To address the problem of the lack of prior knowledge on the target dataset, the initiation stage for accumulating the prior knowledge on the gait is designed for the proposed AGD-SSC method. In the initiation stage, the 1^st^ step in Figure 2(b), the gait images of a certain number of individual walking subjects and the corresponding classification labels (abnormal or normal) assigned by experienced persons based on visual inspections are collected. The number of involved individuals in this stage is specified as “*L*.” To accumulate enough gait data of the individuals for the further classification, no classification result is generated by the proposed AGD-SSC method; in other words, the function of gait anomaly detection is disabled in this stage. When the data accumulation operation is finished, the online anomaly gait detection method (from the 2^nd^ to the 4^th^ steps in Figure 2(b)) starts to run in standard working mode on the foundation of the knowledge on the targeted individual subjects.

The detail of the proposed AGD-SSC method can be elaborated with five sections given below.

### 4.1. Depth Image Acquirement and Selection

**Figure 3:**
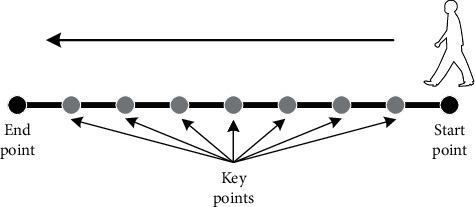
The location points for image selection.

Rather than a measure of intensity or color, pixels in a depth image record depth in the scene. Compared with traditional cameras, depth cameras offer several advantages: working with good invariance against color and texture changes, giving a calibrated scale estimate, and being invariant against illumination level changes. In the image acquirement stage, the side-view images of the individuals are acquired by a depth image camera in the 1^st^ operation of the proposed AGD-SSC method. Each frame of the gait data contains the depth image in the form of the binary image. And, the images are collected automatically with a predefined time interval. To remove the influences of the gait speed differences between the individuals, in the 2nd operation, the frames containing the target individual's complete silhouette are automatically selected from all the collected images. Moreover, to guarantee the numbers of the frames, *N*, for each individual is fixed, the image selection key points of the walking path are defined. It is illustrated in Figure 3 that the virtual key location points projected by the computer are distributed in the walking path evenly and sequentially. In the image acquirement procedure, the number of the camera frames is larger than the number of the key location points, and the captured frames with their centroid closest to a certain key location point in the horizontal direction are selected while the others are discarded.

### 4.2. GEI Generating for Each Individual Subject

The depth images of the selected frames may contain some noise, and part of the background may connect to the body shape silhouette. The silhouettes extracted directly from the images may not be accurate and cannot be used to generate GEI. Therefore, the 3rd operation of the proposed method focuses on removing noise with the two morphological operators, dilation and erosion, in turn. And, the 4th operation of the proposed method is to extract the accurate body shape from the depth image by selecting the largest contour and cutting a proportion of the contour from the bottom to remove the background of the floor that connects to the body shape.

After the silhouettes have been extracted from original walking sequences, the 5^th^ operation is to normalize the size of the silhouette images and align all the silhouette images with their centroids overlapping as indicated in [38]. In the 6^th^ operation, the GEI for an individual is generated with equation (1). It should be noticed that, in the proposed AGD-SSC method, the sequence of the gait silhouette images does not need to reflect the complete gait cycles. It may contain some images corresponding to some complete gait cycles and some images corresponding to some incomplete gait cycles. It meets the requirement of our proposed method as long as the number of the selected images is identical for each subject. In most existing research studies, a key step for the classification methods based on GEI is to identify a complete gait cycle, which costs more computational resources. If the number of the obtained gait sequence is insufficient to constitute a complete gait cycle, the corresponding classification methods will be impractical. Since this step of identifying a complete gait cycle is not required in AGD-SSC, its obvious advantages are the faster execution speed and the wider potential application scenarios.

It is worth mentioning that the first 6 operations discussed so far are necessary for every single subject in the gait anomaly detection procedure. In other words, it is a must to perform the 1^st^ to 6^th^ operations in sequence for each involved subject to produce the essential data for the subsequent operations. And, it should be noticed that the subsequent 3 operations are based on the data of all the involved subjects.

### 4.3. F-GEI Calculation and Gait Features' Extraction

For the gait feature extraction, we define the concept of the F-GEI which stands for Full GEI. The F-GEI is an important constructed image generated in the 7^th^ operation of Figure 2(a), and it is calculated with the equation as follows:

(2) $$\begin{array}{c} F\left(x,y\right)=\frac{1}{M}\overset{M}{\underset{k=1}{\sum }}G_{k}\left(x,y\right), \end{array}$$

where *M* is the number of GEIs generated for the individuals involved in the gait anomaly detection so far. For the incremental online anomaly gait detection scenario, *M* is increased when more individuals are involved.

From equation (2), it can be seen that the F-GEI is the normalized accumulative energy of all the involved individual agents in their walking cycles. It makes use of the idea of GEI and has the power of representing the gait energy distribution of all the involved individual subjects so far.

When an individual subject passes through, the gait data is acquired and then the GEI is generated. The F-GEI can be calculated according to the incremental equation as follows:

(3) $$\begin{array}{c} F_{M}\left(x,y\right)=\frac{1}{M}\left(G_{M}\left(x,y\right)+\left(M-1\right)F_{M-1}\left(x,y\right)\right). \end{array}$$

In the 8^th^ operation of Figure 2(a), the feature vector of each target individual is extracted from the gait data that have been processed in the former 7 operations. Firstly, the F-GEI is transformed into a binary image *E*, which represents the main energy of F-GEI, with the threshold segmentation method given as follows:

(4) $$\begin{array}{c} E\left(x,y\right)=\left\{\begin{array}{cc} 255, & \text{if }F_{M}\left(x,y\right)\geq T,\\ 0, & \text{if }F_{M}\left(x,y\right)<T, \end{array}\right. \end{array}$$

where *T* is the threshold value, which is a predefined value or determined by a discriminating criterion, such as Kapur's entropy optimized with evolutionary algorithms [48], which is used to subdivide the original image into the target object and the background. The pixels with a gray level smaller than *T* are regarded as part of the background, and their gray levels are changed to “0.” The other pixels are regarded as part of the target object and their gray levels are changed to “255.” The target object of the image *E* is representing the high-density area in which the involved individuals are most likely to appear. And then, each element of the vector *o*_*i*_=[*o*_*i*,1_, *o*_*i*,2_,…, *o*_*i*,*N*_] containing features of the *N* frames of the gait cycles of individual *i* is generated with the following equation:

(5) $$\begin{array}{c} o_{i,j}=\frac{\sum_{x=1}^{W}\sum_{y=1}^{H}\left(\left(B_{i,j}\left(x,y\right)==255\right)\times \left(E\left(x,y\right)==255\right)\right)}{\sum_{x=1}^{W}\sum_{y=1}^{H}\left(E\left(x,y\right)==255\right)}, \end{array}$$

where *W* and *H* is the width and height of the images and *E*(*x*, *y*) and *B*_*i*,*j*_(*x*, *y*)  are the gray values of *E* and the *j*th image in the walking sequence of the individual subject *i* in the coordinate (**x**, **y**), respectively. When the conditional expression in equation (5), such as *E*(*x*, *y*)==255, is satisfied, the expression returns 1 and returns 0, otherwise.

From equation (5), we can see that the elements of vector *o*_*i*_ are representing the proportions of the silhouette images in walking sequences overlapping with the *E*. As mentioned in Section 4.2, the sequence of the gait silhouette images of individuals involved does not have to reflect the complete gait cycles in our proposed method. And, the gait periods of the individuals in the same key location points for image selection operation may be different. For example, in a certain image capture key point, an individual is with the double-support stance while another individual is with the legs-together stance. If the vector *o*_*i*_ is used as the feature vector for classification directly, the result will be obviously inaccurate. Therefore, in the final step of this operation, the mean and the standard deviation values of each vector *o*_*i*_ are used as the feature vector of the gait of the *i*th individual subject:

(6) $$\begin{array}{c} v_{i}=\left[\text{mean}\left(o_{i}\right),\text{Std}\left(o_{i}\right)\right]. \end{array}$$

The main goal of feature selection is to improving classification accuracy [49]. By choosing the mean and the standard deviation values of the vector o_*i*_ to present the gait features, the feature extraction method has two advantages. (1) Since the silhouette noise in individual frames and the noise caused by the individuals' different stances in the same location are filtered out, the feature extraction method is less sensitive to noise. (2) Since the feature vector definition conforms to the basis of the proposed AGD-SSC method, that is, the similarity between normal gaits is statistically high, while the similarity between abnormal gait and normal gait is statistically low, it is beneficial for subsequent classifiers to obtain accurate classification result.

### 4.4. The Constrained *K*-Means Clustering Algorithm

Since few labeled samples are known in advance on the online anomaly gait detection scenarios targeted by the proposed AGD-SSC method, those classification methods that require a large number of training samples are impossible to be adopted by this study. The main benefit of semisupervised clustering algorithms is improving clustering results using limited supervision, which exactly matches the requirement of the target application scenarios. Therefore, the classification operation of the proposed AGD-SSC method, the 9^th^ operation in Figure 2(a), is performed by an improved COP-*K*-means algorithm.

All machine learning methods work with specific assumptions. The key assumption of our proposed method is that the deviation from the normal gait baseline pattern indicates a possible gait anomaly. This assumption has been verified in some earlier research studies based on both the nonmachine learning approach [50] and the machine learning approach [15]. In our observation, by representing the mean and the standard deviation values of the proportions of the silhouette images in walking sequences overlapping with the image *E*, the feature vectors of the abnormal gait sequences resembled each other and thereby can be classified as a single class. Therefore, all the individuals' gaits can be classified into two clusters: one is an abnormal gait class and the other is a normal gait class. Based on the known classification labels (abnormal or normal) of some individual subjects, the member in one of the clusters can be judged as individuals with normal gait patterns, while the members of the other cluster are regarded as individuals with abnormal gait patterns.

Although many variants of the COP-K-means algorithm have been proposed, however, as this was not the main objective in our present study, we did not investigate these variants and only developed our modified algorithm based on the basic form of the COP-K-means algorithm as the classifier. The pseudocode of the COP-*K*-means algorithm is given in Algorithm 1, and more details of its idea and advantage can be found in Section 3.2.

The number of clusters *K* in the *K*-means clustering algorithm is assumed to be predefined. In the online anomaly gait detection, especially in the early stage, all the involved individual subjects maybe with normal gaits and thereby at the same single class. Therefore, if the COP-K-means algorithm is directly used to classify the individuals by their gait features, the misclassification may occur because of the inherent characteristic of the clustering algorithm. Combining a novel mechanism developed in this study called boundary clamping, a modified constrained clustering algorithm called BC-COP-*K*-means, which stands for Boundary Clamping COP-*K*-means, is proposed to handle this problem.

The proposal of the BC-COP-*K*-means algorithm is mainly based on two motivations. (1) The labeled data samples are the important resources for the semisupervised clustering algorithm, and full use of the information of labeled data samples can improve the accuracy of the semisupervised clustering algorithm. (2) Another prior knowledge on the target application scenario, that is, the dataset can only be clustered into two classes, can be also used to improve the performance of the algorithm. The boundary clamping operation is applied to the new involved individual subject based on prior knowledge about the dataset. The main idea of the boundary clamping is that if the feature values of a new individual are all within the boundary values derived from the feature values of the labeled samples in a certain cluster, then it should be a member of the same cluster. To avoid reducing the accuracy of classification, the individual walking subjects involved in calculating the boundary values of the cluster must be the subjects with labels known in advance. Other than being used as the constraint for the clustering, the information of the labeled samples is used in the boundary clamping operation to help determine the class of data points.

The modified k-means algorithm (BC-COP-*K*-means) is illustrated in Algorithm 2. Compared with the direct K-means clustering algorithm, there is an extra input dataset containing disjoint *λ* sets of data points with preassigned class labels {*Q*_*p*_}_*p*=1_^*λ*^. Each subset *Q*_*p*_ has the inherent and implicit information that each member has the same class label *p*. Before the COP-*K*-means algorithm can be run, the pairwise constraints containing must-link constraints and cannot-link constraints must be generated according to the datasets {*Q*_*p*_}_*p*=1_^*λ*^. The pairwise constraints generating procedure is illustrated in Algorithm 3. Any two data points from the dataset {*Q*_*p*_}_*p*=1_^*λ*^ are picked and checked. If the two data points are from the same subset, which means they belong to the same cluster, a must-link constraint is generated, and a cannot-link constraint is generated otherwise.

It can be seen that the proposed BC-COP-*K*-means algorithm extends the original COP-*K*-means algorithm based on an additional assumption about the dataset. Before the COP-*K*-means algorithm is run, the boundary clamping operation is used to determine whether a further operation should be performed. When the new involved individual subject can be classified into one of the clusters by boundary clamping operation, the subsequent clustering operation will be ignored. One of the main advantages of the BC-COP-*K*-means algorithm is that it can make use of prior knowledge in multiple aspects to improve classification accuracy. On the contrary, no parameter is needed to be set for the BC-COP-*K*-means algorithm, which implies easy implementation and wide application prospects. Moreover, the complexity of the boundary clamping operation is significantly lower than that of *K*-means clustering. Therefore, the proposed BC-COP-*K*-means algorithm has a potential higher computational efficiency than the original COP-*K*-means algorithm.

### 4.5. Classification

The pseudocode of the gait classification is given in Algorithm 4. It is clear that the classification based on the proposed BC-COP-K-means algorithm is straightforward. The proposed AGD-SSC method can start producing detection results under the work of only collecting a tiny number of gait samples, even though none of those gait samples are abnormal. Therefore, the parameter *λ* equals 1 if the set of prelabeled samples contain only one class and equals 2 otherwise.

## 5. Experimental Validation

Experiments are carried out to verify the effectiveness of the proposed AGD-SSC method under the tuning on different prior knowledge about the dataset. Moreover, performance is evaluated on classification accuracy, stability, and computation efficiency of the proposed method in comparison to other state-of-the-art anomaly detection methods, including Isolation Forest (IF) [28] and Local Outlier Factor (LOF) [27]. Both the IF and LOF are regarded as efficient anomaly detection methods and are almost always among those methods presented at rankings in the literature. For the experiment, the IF and LOF are run under the same flow as the AGD-SSC method with replacing the proposed BC-COP-*K*-means method in the classification stage. In addition, to evaluate the effectiveness of the boundary clamping mechanism, one more method is designed for comparison. The method is called AGD-SSC-NBC, which stands for the AGD-SSC method without the boundary clamping operation. The AGD-SSC-NBC method has the same flow as the AGD-SSC method, except the clustering algorithm adopted is the direct COP-K-means.

All the compared methods are coded with Python 3, and the experiments on the IF and LOF algorithms are based on the implementation provided by the scikit-learn library. In realistic application scenarios, the information of the data about to process, such as the proportion of abnormal data, is normally implicitly unavailable. Therefore, the corresponding setting of the algorithms' parameters can only be set with the predefined values empirically. In the experiment, there is no parameter needed to set the AGD-SSC method and the AGD-SSC-NBC, while the parameters for the IF and the LOF were set as with the default values in scikit-learn 0.19.1. Actually, when the IF and the LOF are applied in our target application, both the algorithms cannot meet the implicit requirement of no parameter should be set. The performances of the IF and the LOF depend on the setting of the corresponding parameters, and it is obviously impossible to know the best parameters of the algorithms for the target application scenario in advance. So, the IF and the LOF are actually not among the favorable choices for our target application. However, in order to provide a comparison of the proposed method, we directly apply the default parameters in scikit-learn on the IF and the LOF algorithms to obtain the results.

### 5.1. Dataset Collection

For realistic gait anomaly detection applications, it is better to collect and use the data of individuals who are actually with an abnormal gait. However, collecting a number of such data is difficult. To simulate an application scenario in the real world, spurious lesions were given to the body parts of some of the subjects by bandaging or immobilizing them. Besides, the natural gait data of some other subjects are also collected. All of the gait data were observed in a laboratory setup in the same test site with the same settings and used to build the test gait dataset. In a certain experiment, the maximum value of *M* is defined as *S*, which refers to the final number of the individuals involved in the incremental online anomaly gait detection. The gait dataset consists of the gait data of 62 young adults (13 females and 49 males) with ages from 20 to 23 walking in a straight path in front of the camera. That means the parameter *S* is 62 in the experiment. 12 of them were spurious lesions by bandaging or immobilizing them while others are in their natural states. The number of gait silhouette images sequence for each individual, the parameter *N*, is set with 16.

**Figure 4:**
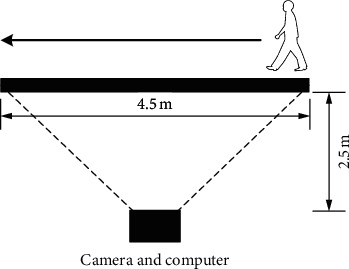
The experimental scene.

For the experiment, the depth camera D435 from Intel Corp. and a personal computer with 3.40 GHz CPU and 8 GB RAM under Windows 10 system are used to acquire and process the data to build the dataset. For data acquisition, the participators went through the walking path in turn in front of the camera. The experiment scene is illustrated in Figure 4. For convenience, we call the participators with spurious lesions as team A and the participators with natural gaits as team B. We use the codes A1, A2, etc. to refer to the members of team A, and the codes B1, B2, etc. to refer to the members of team B.

**Figure 5:**
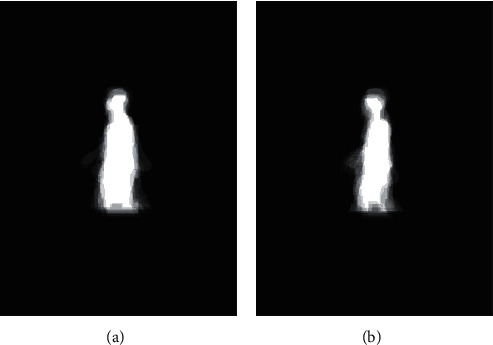
The GEIs of normal gait.

**Figure 6:**
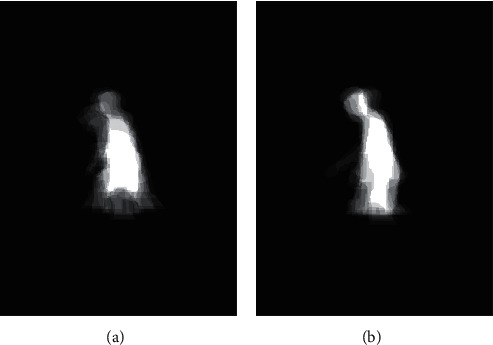
The GEIs of abnormal gait.

When each participator passes through the walking path, the images are captured, and the operations from the 1^st^ operation to the 6^th^ operation in Figure 2(a) are used to generate the *N* frames of the normalized gait images and the corresponding GEIs. Two GEI samples of the normal gait are illustrated in Figure 5. In the meantime, two GEI samples of the abnormal gait are given in Figure 6.

### 5.2. Test Cases

For our target online anomaly gait detection scenario, except for the initiation stage, the feature extraction and classification operations are performed when each individual passes through the camera. It can be seen that the feature vector of each individual is determined by the gait image of itself and the current F-GEI. And, the current F-GEI is determined by the accumulation of the images of the current tested individual and the individuals who have been tested. Therefore, the sequence of the individuals who went through the walking path has an important impact on the classification results. After the *N* frames of the normalized gait images and the corresponding GEIs is acquired, we run the experiment with three test cases as follows. (I) The test sequence indicating the order of the individuals is generated, and the gait anomaly detection is performed from the 5th individual in the test sequence, which means that the parameter *L* in Figure 2(b) is set to 4 in this test case. (II) The gait anomaly detection tasks with the parameter *L* are set as 6. (III) The gait anomaly detection tasks with the parameter *L* are set as 8.

It is more likely that the individuals passing through the camera in a random sequence in the application scenario. To meet the situation of the application in the real world and to evaluate the stability of the proposed method, the classification tasks in the experiment are run under 10 different randomly generated sequences of the individuals, as illustrated in Table 1. Each test sequence is listed in two columns in the table, and the left column is the first half of the sequence. The members in team A are highlighted with a gray background. It can be seen that the individuals are distributed uniformly in the test sequences and each test sequence can represent a typical situation of the applications in the real world. Moreover, the different settings on parameter *L* for the three test cases are to evaluate the impact of the number of labeled samples, which reflects the amount of prior knowledge about the dataset, to the proposed method.

Moreover, the proposed AGD-SSC method is based on *K*-means, which is known to be sensitive to different initializations. Therefore, each sequence in each test case was run for 30 trials, and the results were shown by their average and standard deviation values. The classification tasks based on the four compared algorithms are implemented in a personal computer with 2.30 GHz CPU and 64 GB RAM under Windows 10 system.

### 5.3. Evaluation Metrics

The online anomaly gait detection result is evaluated by comparing the obtained label of each individual's gait with that known. The metric, correct classification rate (CCR) [51], is used to measure the classification performance of the proposed method in the experiment. Let *a*_*i*_ and *b*_*i*_ be the obtained label and the known label of the *i*th gait in each test sequence, respectively. CCR in this experiment is defined as follows:

(7) $$\begin{array}{c} \text{CCR}=\sum_{i=L+1}^{S}\frac{\delta \left(a_{i},b_{i}\right)}{\left(S-L\right)},\; \end{array}$$

where *S* is the total number of the individuals in a classification task, *L* is the number of labeled samples as mentioned previously, and *δ*(*a*, *b*) is the delta function that equals 1 if *a*=*b* and equals 0 otherwise. It is obvious that the metric CCR is for a whole online anomaly gait detection procedure from the (*L+1)*th individual to the *S*th individual.

In gait anomaly detection, the main target is to find out the individuals with abnormal gaits. If individuals with an abnormal gait are misclassified as the one with normal gait sequences, in other words, the abnormal gait is not detected, the consequence may be severe. For example, in the application of body condition detection for the elderly in a nursing home, the failure of abnormal gait detection means the missing of detecting the potential disease of the elderly in the early stage, which may lead to the deteriorated condition. Therefore, a metric, anomaly correct classification rate (ACCR), is defined to illustrate the capability of the algorithm to detect the abnormal gait. The equation for calculating ACCR is as follows:

(8) $$\begin{array}{c} \text{ACCR}=\sum_{i=1}^{Z}\frac{\delta \left(a_{u_{i}},b_{u_{i}}\right)}{Z}, \end{array}$$

where *u*_*i*_ ∈ *U*, *U*={*u*_1_, *u*_2_, ⋯, *u*_*Z*_} is the set containing all the items of the abnormal gait of the dataset in the experiment, and *Z*=|*U*| is the number of elements in the set *U*.

### 5.4. Experimental Results

The classification accuracy and stability and computation efficiency of the proposed AGD-SSC method in comparison to the other algorithms will be presented in the subsequent sections.

#### 5.4.1. Classification Accuracy and Stability

To illustrate the classification accuracy and stability of the proposed AGD-SSC method, the mean and standard deviation of CCR and ACCR values obtained in the 30 independent trials on the 10 test sequences of the three test cases by the proposed AGD-SSC method, AGD-SSC-NBC method, IF method, and LOF method are given in Tables 2–4, respectively. The total CCR and ACCR values of the three test cases are also shown in the corresponding tables. Obviously, a higher mean value of the metric values indicates higher classification accuracy while a lower standard deviation value represents better stability. The best results among those obtained by all four contenders for each test sequence of each test case are highlighted in boldface.

The results in Tables 2–4 reveal that the proposed AGD-SSC method obtains the best mean values in 18 out of the 20 instances with the least standard deviation in 14 out of the 20 instances in the test case I, obtains the best mean values in 18 out of the 20 instances with the least standard deviation in 14 out of the 20 instances in the test case II, and obtains the best mean values in all of the 20 instances with the least standard deviation in 17 out of the 20 instances in the test case III. It shows that the proposed BC-COP-*K*-means method outperforms all the compared algorithms in solution accuracy. Due to the randomness of the cluster centers' initialization for the proposed AGD-SSC method, its stability is not the best among the compared algorithms. However, the standard deviation values of most instances in the experiment are zero or close to zero. It indicates the proposed AGD-SSC method is capable of producing stable results.

Moreover, in test case I, the proposed AGD-SSC method achieves a total CCR of 95.54%. After increasing the amount of prior knowledge about the dataset, the overall CCR of the proposed AGD-SSC method improves to 95.91% and 96.69% in test case II and test case III, respectively. It indicates that more prior knowledge is beneficial for improving the total classification accuracy. Meanwhile, the total ACCR values of the three test cases are similar to each other, and it indicates that the ACCR results are not sensitive to the change of the amount of prior knowledge.

From the compared results of the proposed AGD-SSC method and the AGD-SSC-NBC method, it can be seen that the proposed AGD-SSC method obtains the better mean values on CCR in 22 out of the 30 instances in all of the three test cases. And, for the total CCR results, the proposed AGD-SSC method outperforms the AGD-SSC-NBC method in all of the three test cases. It is evident that the AGD-SSC has a more accurate classification ability empowered by the boundary clamping mechanism.

#### 5.4.2. Computation Efficiency

In addition to classification accuracy, short finite computation time is a must for the method in real-time applications. The CPU time (measured in seconds) by all the four contenders on each test instance is shown in Tables 5–7, respectively. Generally, compared with the AGD-SSC-NBC method, the proposed AGD-SSC method obtains the smaller mean values on all of the 30 instances. It is clear that the proposed AGD-SSC method has a high and stable computational efficiency empowered by the boundary clamping mechanism. Although the LOF algorithm outperforms our proposed AGD-SSC method in running speed, the classification results obtained by the LOF algorithm are not satisfactory. Therefore, combining the classification accuracy and stability and computation efficiency, it can be concluded that the overall performance of the proposed AGD-SSC method is the best amongst the four contenders.

## 6. Conclusion and Future Work

In this paper, we propose a novel practical and efficient automatic gait anomaly detection method that is running in an online manner to produce the binary classification result on the gait of each individual passes through the test point. In the proposed AGD-SSC method, a new concept of the F-GEI is introduced which is the normalized accumulative energy of all the involved individual subjects in their walking cycles. The deviation of the silhouette images in walking sequences of each individual to the main energy of the F-GEI is used to represent gait features in the form of two elements tuple. Furthermore, combining the newly designed boundary clamping mechanism, an improved constraint K-means clustering algorithm called BC-COP-*K*-means is proposed to classify the gaits to abnormal gait class or the normal gait class with the extracted feature vectors.

Experiments are conducted on the human gaits' dataset containing both normal gait data and abnormal gait data. Three test cases with different numbers of pre-known labeled samples are designed. Experimental results have shown that the proposed BC-COP-K-means algorithm is an efficient and effective binary classification technique that outperforms three state-of-the-art anomaly detection techniques, including IF, LOF, and direct COP-K-means. From the experiment, it can be deduced that the proposed AGD-SSC method is potentially efficient in automatic gait anomaly detection for humans when their clothing and shoes are not very diverse to ensure that only slight silhouette distortion is brought. Moreover, since the proposed method is not sensitive to the body shape of the target subjects, it is also a potentially promising solution for the gait anomalies of the large farming animals such as cattle, pigs, and sheep whose gaits are naturally not affected by clothing or shoes.

If the clothing and shoes are dramatically diverse for individual people, some other methods such as the efficient templates producing method need to be combined to improve the classification accuracy. And that is one of our future works. Another aspect that remains to be investigated in our future work is to design practical systems with embedded processors to carry out experiments with the AGD-SSC method on some applications such as alcohol usage alert in the parking lots.

## Figures and Tables

**Algorithm 1:**
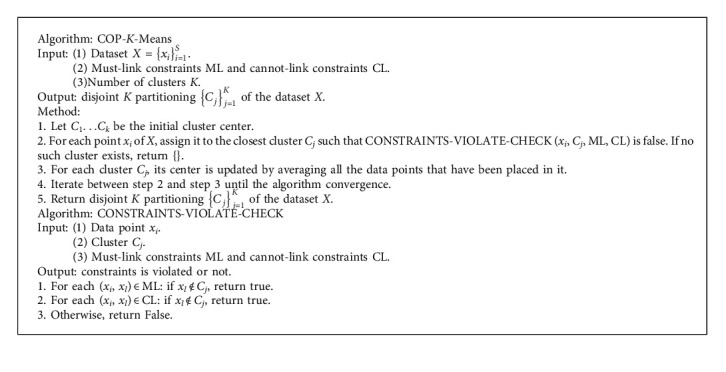
The pseudocodes of the COP-*K*-means algorithm.

**Algorithm 2:**
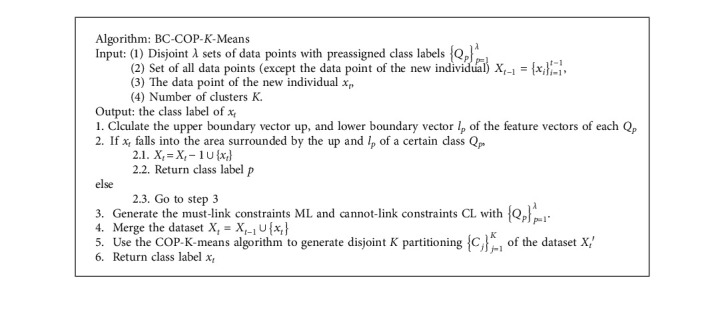
The pseudocodes of the BC-COP-*K*-means algorithm.

**Algorithm 3:**
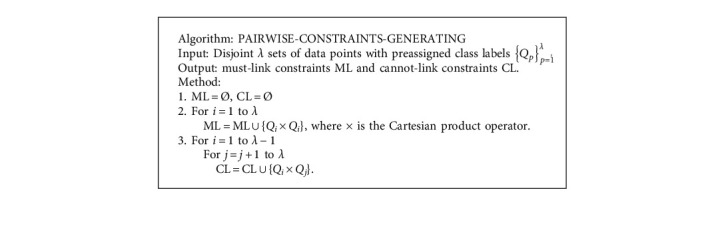
The pseudocodes of the pairwise constraint generating.

**Algorithm 4:**
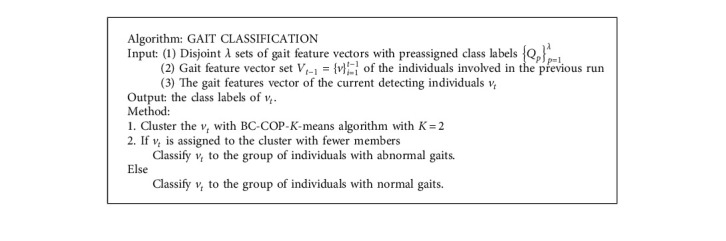
The pseudocodes of the gait classification.

**Table 1:** The sequences of the individuals for experiment.

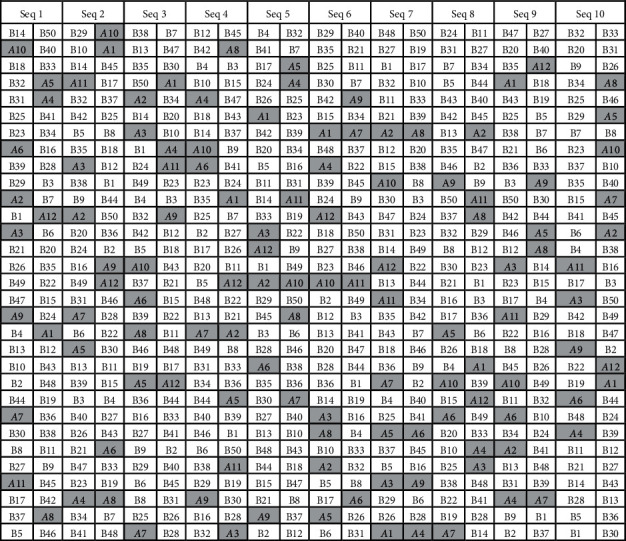

**Table 2:** CCR/ACCR values for AGD-SSC, AGD-SSC-NBC, IF, and LOF in test case I.

Seq. No.	Metric	AGD-SSC mean (st. dev.)	AGD-SSC-NBC mean (st. dev.)	IF mean (st. dev.)	LOF mean (st. dev.)
Seq. 1	CCR	**0.9138 (0.0000)**	**0.9138 (0.0000)**	0.8897 (0.0105)	0.8793 **(0.0000)**
	ACCR	**0.9167 (0.0000)**	**0.9167 (0.0000)**	0.5333 (0.0408)	0.5000 **(0.0000)**

Seq. 2	CCR	0.9138 **(0.0000)**	0.9132 (0.0031)	**0.9328** (0.0052)	0.8621 **(0.0000)**
	ACCR	**1.0000 (0.0000)**	**1.0000 (0.0000)**	0.7583 (0.0250)	0.4167 **(0.0000)**

Seq. 3	CCR	**0.9828 (0.0000)**	**0.9828 (0.0000)**	0.8822 (0.0064)	0.8793 **(0.0000)**
	ACCR	**0.9167 (0.0000)**	**0.9167 (0.0000)**	0.5139 (0.0311)	0.4167 **(0.0000)**

Seq. 4	CCR	**0.9529** (0.0076)	0.9500 (0.0081)	0.8902 (0.0169)	0.8793 **(0.0000)**
	ACCR	**0.9167 (0.0000)**	**0.9167 (0.0000)**	0.5389 (0.0826)	0.5833 **(0.0000)**

Seq. 5	CCR	0.9310 **(0.0000)**	0.8966 **(0.0000)**	**0.9408** (0.0139)	0.8793 **(0.0000)**
	ACCR	**0.9167 (0.0000)**	**0.9167 (0.0000)**	0.7333 (0.0500)	0.4167 **(0.0000)**

Seq. 6	CCR	**0.9828 (0.0000)**	0.9310 **(0.0000)**	0.8874 (0.0139)	0.8793 **(0.0000)**
	ACCR	**0.9167 (0.0000)**	**0.9167 (0.0000)**	0.5694 (0.0573)	0.5000 **(0.0000)**

Seq. 7	CCR	**0.9644** (0.0043)	0.9471 (0.0043)	0.9052 (0.0097)	0.9310 **(0.0000)**
	ACCR	**0.9944** (0.0208)	**0.9944** (0.0208)	0.6278 (0.0416)	0.6667 **(0.0000)**

Seq. 8	CCR	**0.9661** (0.0083)	0.9103 (0.0103)	0.9109 (0.0167)	0.8793 **(0.0000)**
	ACCR	**0.9167** (0.0215)	**0.9167 (0.0215)**	0.6722 (0.0643)	0.5833 **(0.0000)**

Seq. 9	CCR	**0.9828 (0.000)**	0.8966 **(0.0000)**	0.8879 (0.0139)	0.8966 **(0.0000)**
	ACCR	**1.0000 (0.0000)**	**1.0000 (0.0000)**	0.8889 (0.0393)	0.7500 **(0.0000)**

Seq. 10	CCR	**0.9638** (0.0052)	0.9310 (0.0000)	0.9511 (0.0141)	0.8966 **(0.0000)**
	ACCR	**1.0000 (0.0000)**	**1.0000 (0.0000)**	0.7861 (0.0556)	0.6667 **(0.0000)**

Total	CCR	**0.9554**	0.9272	0.9078	0.8862
	ACCR	**0.9494**	**0.9494**	0.661	0.5500

**Table 3:** CCR/ACCR values for AGD-SSC, AGD-SSC-NBC, IF, and LOF in test case II.

Seq. No.	Metric	AGD-SSC mean (st. dev.)	AGD-SSC-NBC mean (st. dev.)	IF mean (st. dev.)	LOF mean (st. dev.)
Seq. 1	CCR	**0.9286 (0.000)**	**0.9286 (0.000)**	0.8869 (0.0106)	0.8929 **(0.0000)**
	ACCR	**0.9167 (0.000)**	**0.9167 (0.0000)**	0.5444 (0.0416)	0.5000 **(0.0000)**

Seq. 2	CCR	0.9119 (0.0045)	0.9125 (0.0071)	**0.9327** (0.0088)	0.8571 **(0.0000)**
	ACCR	**1.0000 (0.0000)**	**1.0000 (0.0000)**	0.7611 (0.0356)	0.4167 **(0.0000)**

Seq. 3	CCR	0.9815 (0.0035)	**0.9821 (0.0000)**	0.8810 (0.0084)	0.8929 **(0.0000)**
	ACCR	**0.9167 (0.0000)**	**0.9167 (0.0000)**	0.5278 (0.0084)	0.5000 **(0.0000)**

Seq. 4	CCR	**0.9482** (0.0071)	0.9476 (0.0064)	0.8839 (0.0120)	0.8929 **(0.0000)**
	ACCR	**0.9167 (0.0000)**	**0.9167 (0.0000)**	0.5361 (0.0513)	0.6667 **(0.0000)**

Seq. 5	CCR	**0.9464 (0.0000)**	0.8393 **(0.0000)**	0.9363 (0.0136)	0.8750 **(0.0000)**
	ACCR	**0.9167 (0.0000)**	**0.9167 (0.0000)**	0.7194 (0.0504)	0.4167 **(0.0000)**

Seq. 6	CCR	**0.9821 (0.0000)**	0.9464 **(0.0000)**	0.8905 (0.0110)	0.8750 **(0.0000)**
	ACCR	**0.9167 (0.0000)**	**0.9167 (0.0000)**	0.5722 (0.0515)	0.5000 **(0.0000)**

Seq. 7	CCR	**0.9643 (0.0000)**	0.9625 (0.0084)	0.9006 (0.0100)	0.9286 **(0.0000)**
	ACCR	**1.0000 (0.0000)**	0.9889 (0.0356)	0.6222 (0.0416)	0.6667 **(0.0000)**

Seq. 8	CCR	**0.9643** (0.0080)	0.9387 (0.0110)	0.9083 (0.0089)	0.8929 **(0.0000)**
	ACCR	**0.9167** (0.0215)	0.9139 (0.0150)	0.6694 (0.0402)	0.5833 **(0.0000)**

Seq. 9	CCR	**0.9821 (0.0000)**	0.9286 **(0.0000)**	0.9036 (0.0182)	0.8929 **(0.0000)**
	ACCR	**1.0000 (0.0000)**	**1.0000 (0.0000)**	0.8972 (0.0466)	0.7500 **(0.0000)**

Seq. 10	CCR	**0.9815** (0.0032)	0.9452 (0.0045)	0.9446 (0.0141)	0.9286 **(0.0000)**
	ACCR	**1.0000 (0.0000)**	**1.000 (0.0000)**	0.7694 (0.0513)	0.6667 **(0.0000)**

Total	CCR	**0.9591**	0.9332	0.9068	0.8929
	ACCR	**0.9500**	0.9486	0.6619	0.5667

**Table 4:** CCR/ACCR values for AGD-SSC, AGD-SSC-NBC, IF, and LOF in test case III.

Seq. No.	Metric	AGD-SSC mean (st. dev.)	AGD-SSC-NBC mean (st. dev.)	IF mean (st. dev.)	LOF mean (st. dev.)
Seq. 1	CCR	**0.9444 (0.0000)**	**0.9444 (0.0000)**	0.8821 (0.0089)	0.9074 **(0.0000)**
	ACCR	**0.9167 (0.0000)**	**0.9167 (0.0000)**	0.5222 (0.0369)	0.5833 **(0.0000)**

Seq. 2	CCR	**0.9815 (0.0000)**	0.9599 (0.0069)	0.9272 (0.0046)	0.8519 **(0.0000)**
	ACCR	**1.0000 (0.0000)**	0.9861 (0.0311)	0.7528 (0.015)	0.4167 **(0.0000)**

Seq. 3	CCR	**0.9815 (0.0000)**	**0.9815 (0.0000)**	0.8920 (0.0097)	0.8889 **(0.0000)**
	ACCR	**0.9167 (0.0000)**	**0.9167 (0.0000)**	0.5972 (0.0435)	0.5000 **(0.0000)**

Seq. 4	CCR	**0.9284** (0.0063)	0.9265 (0.0033)	0.8988 (0.0149)	0.8889 **(0.0000)**
	ACCR	**0.9167 (0.0000)**	**0.9167 (0.0000)**	0.6167 (0.0593)	0.6667 **(0.0000)**

Seq. 5	CCR	**0.9444 (0.0000)**	0.8704 **(0.0000)**	0.9364 (0.0124)	0.8704 **(0.0000)**
	ACCR	**0.9167 (0.0000)**	**0.9167 (0.0000)**	0.7278 (0.0472)	0.4167 **(0.0000)**

Seq. 6	CCR	**0.9815 (0.0000)**	0.9630 **(0.0000)**	0.8796 (0.0104)	0.8889 **(0.0000)**
	ACCR	**0.9167 (0.0000)**	**0.9167 (0.0000)**	0.5417 (0.0469)	0.5000 **(0.0000)**

Seq. 7	CCR	**0.9630 (0.0000)**	**0.9630 (0.0000)**	0.8938 (0.0106)	0.9444 **(0.0000)**
	ACCR	**1.000 (0.0000)**	**1.0000 (0.0000)**	0.6111 (0.0393)	0.7500 **(0.0000)**

Seq. 8	CCR	**0.9809** (0.0033)	0.9549 (0.0092)	0.9056 (0.0120)	0.8889 **(0.0000)**
	ACCR	**09139** (0.0150)	**0.9139** (0.0150)	0.6583 (0.0542)	0.5833 **(0.0000)**

Seq. 9	CCR	**0.9815 (0.0000)**	0.9444 **(0.0000)**	0.9019 (0.0137)	0.8889 **(0.0000)**
	ACCR	**1.0000 (0.0000)**	**1.0000 (0.0000)**	0.9000 (0.0333)	0.7500 **(0.0000)**

Seq. 10	CCR	**0.9815 (0.0000)**	0.9617 (0.0046)	0.9451 (0.0155)	0.9259 **(0.0000)**
	ACCR	**1.0000 (0.0000)**	**1.0000 (0.0000)**	0.7889 (0.0468)	0.6667 **(0.0000)**

Total	CCR	**0.9669**	0.9470	0.9062	0.8944
	ACCR	**0.9497**	0.9483	0.6717	0.5833

**Table 5:** The CPU timing of the test case I by the AGD-SSC, AGD-SSC-NBC, IF, and LOF.

Seq no.	AGD-SSC mean (st. dev.)	AGD-SSC-NBC mean (st. dev.)	IF mean (st. dev.)	LOF mean (st. dev.)
Seq. 1	2.0163 (0.0356)	2.2827 (0.0387)	11.1441 (0.0641)	0.9372 (0.0103)
Seq. 2	1.2471 (0.0282)	3.1222 (0.0534)	11.1497 (0.0683)	0.9472 (0.0283)
Seq. 3	1.3787 (0.0307)	2.4007 (0.0165)	11.1502 (0.0742)	0.9482 (0.0293)
Seq. 4	1.8787 (0.00353)	2.2930 (0.0243)	11.1385 (0.0365)	0.9398 (0.0079)
Seq. 5	1.3027 (0.0282)	2.3197 (0.0859)	11.1385 (0.0150)	0.9437 (0.0127)
Seq. 6	1.2168 (0.0328)	2.3275 (0.0376)	11.2123 (0.2133)	0.9451 (0.0282)
Seq. 7	1.5393 (0.0502)	2.3225 (0.0255)	11.1698 (0.0941)	0.9481 (0.0285)
Seq. 8	1.2986 (0.0387)	2.0971 (0.0265)	11.1423 (0.0682)	0.9442 (0.234)
Seq. 9	1.4610 (0.0360)	2.2503 (0.0253)	11.1374 (0.0165)	0.9509 (0.0376)
Seq. 10	1.4347 (0.0544)	2.2272 (0.0250)	11.2059 (0.2808)	0.9484 (0.0359)

Average	1.4774	2.364	11.1589	0.9453

**Table 6:** The CPU timing of the test case II by the AGD-SSC, AGD-SSC-NBC, IF, and LOF.

Seq no.	AGD-SSC mean (st. dev.)	AGD-SSC-NBC mean (st. dev.)	IF mean (st. dev.)	LOF mean (st. dev.)
Seq. 1	1.8829 (0.0198)	2.1767 (0.0168)	10.7176 (0.0397)	0.9176 (0.0064)
Seq. 2	1.6232 (0.0606)	2.8794 (0.0934)	10.7297 (0.0596)	0.9319 (0.0280)
Seq. 3	1.2637 (0.0333)	2.3155 (0.0365)	10.7345 (0.0681)	0.9293 (0.0224)
Seq. 4	1.7210 (0.0357)	2.2155 (0.0327)	10.7328 (0.0721)	0.9261 (0.0203)
Seq. 5	1.1815 (0.0096)	2.1598 (0.0625)	10.7578 (0.2188)	0.9231 (0.0108)
Seq. 6	1.1071 (0.0366)	2.2243 (0.0303)	10.7458 (0.0954)	0.9265 (0.0231)
Seq. 7	1.3799 (0.0291)	2.0953 (0.0304)	10.7269 (0.0342)	0.9274 (0.0214)
Seq. 8	1.2086 (0.0247)	2.0500 (0.0354)	10.7376 (0.0957)	0.9272 (0.0229)
Seq. 9	1.3493 (0.0152)	2.1763 (0.0381)	10.7269 (0.0455)	0.9248 (0.0062)
Seq. 10	1.2157 (0.0347)	2.1177 (0.0327)	10.7687 (0.1951)	0.9276 (0.0251)

Average	1.3933	2.2411	10.7378	0.9262

**Table 7:** The CPU timing of the test case III by the AGD-SSC, AGD-SSC-NBC, IF, and LOF.

Seq. no.	AGD-SSC mean (st. dev.)	AGD-SSC-NBC mean (st. dev.)	IF mean (st. dev.)	LOF mean (st. dev.)
Seq. 1	1.3630 (0.0198)	2.0991 (0.0188)	10.3548 (0.0274)	0.8779 (0.0093)
Seq. 2	0.9329 (0.0230)	2.4315 (0.0703)	10.3604 (0.0427)	0.8888 (0.0249)
Seq. 3	1.2131 (0.0135)	2.2532 (0.0214)	10.3636 (0.0170)	0.8875 (0.0222)
Seq. 4	1.6834 (0.0280)	2.1603 (0.0303)	10.3877 (0.1105)	0.8844 (0.0228)
Seq. 5	1.1467 (0.0233)	2.0710 (0.0313)	10.3720 (0.0779)	0.8873 (0.0290)
Seq. 6	1.0755 (0.0486)	2.1199 (0.0306)	10.4088 (0.2120)	0.8901 (0.0459)
Seq. 7	1.3052 (0.0413)	3.5250 (0.1036)	10.3717 (0.0829)	0.8869 (0.0287)
Seq. 8	1.0829 (0.0356)	0.9906 (0.0323)	10.3682 (0.0704)	0.8866 (0.0271)
Seq. 9	1.3358 (0.0961)	2.1020 (0.0131)	10.3679 (0.0712)	0.8900 (0.0336)
Seq. 10	1.1698 (0.0552)	2.0080 (0.0292)	10.3664 (0.0693)	0.8884 (0.0264)

Average	1.2308	2.2761	10.3721	0.8868

## Data Availability

The data used to support the findings of this study are available from the corresponding author upon request.

## References

[1] Horn C., Willett R. Online anomaly detection with expert system feedback in social networks.

[2] Troje N. F. (2002). Decomposing biological motion: a framework for analysis and synthesis of human gait patterns. *Journal of Vision*.

[3] Pogorelc B., Bosnić Z., Gams M. (2012). Automatic recognition of gait-related health problems in the elderly using machine learning. *Multimedia Tools and Applications*.

[4] Liston R., Mickelborough J., Bene J., Tallis R. (2003). A new classification of higher level gait disorders in patients with cerebral multi-infarct states. *Age and Ageing*.

[5] Su F.-C., Wu W.-L., Cheng Y.-M., Chou Y.-L. (2001). Fuzzy clustering of gait patterns of patients after ankle arthrodesis based on kinematic parameters. *Medical Engineering & Physics*.

[6] Kao H.-L. C., Ho B.-J., Lin A. C., Chu H.-H. Phone-based gait analysis to detect alcohol usage.

[7] Zhu W. X., Wang J. P., Zhu J. J. (2014). Walking posture analysis of pigs based on star skeleton model. *Applied Mechanics and Materials*.

[8] Song X., Leroy T., Vranken E., Maertens W., Sonck B., Berckmans D. (2008). Automatic detection of lameness in dairy cattle-Vision-based trackway analysis in cow’s locomotion. *Computers and Electronics in Agriculture*.

[9] Pluk A. (2010). Evaluation of step overlap as an automatic measure in dairy cow locomotion. *Transactions of the ASABE*.

[10] Zhao K., He D. Real-time automatic classification of lameness in dairy cattle based on movement analysis with image processing technique.

[11] Alaqtash M., Sarkodie-Gyan T., Yu H., Fuentes O., Brower R., Abdelgawad A. Automatic classification of pathological gait patterns using ground reaction forces and machine learning algorithms.

[12] Saho K., Fujimoto M., Masugi M., Chou L.-S. (2017). Gait classification of young adults, elderly non-fallers, and elderly fallers using micro-Doppler radar signals: simulation study. *IEEE Sensors Journal*.

[13] Manap H. H., Tahir N. M., Yassin A. I. M. Anomalous gait detection based on support vector machine.

[14] Cola G., Avvenuti M., Vecchio A., Yang G.-Z., Lo B. (2015). An on-node processing approach for anomaly detection in gait. *IEEE Sensors Journal*.

[15] Haladjian J., Hodaie Z., Nüske S., Brügge B. Gait anomaly detection in dairy cattle.

[16] Pau M., Mandaresu S., Pilloni G. (2017). Smoothness of gait detects early alterations of walking in persons with multiple sclerosis without disability. *Gait & Posture*.

[17] Alaqtash M., Yu H., Brower R., Abdelgawad A., Sarkodie-Gyan T. (2011). Application of wearable sensors for human gait analysis using fuzzy computational algorithm. *Engineering Applications of Artificial Intelligence*.

[18] Jones L., Beynon M. J., Holt C. A., Roy S. (2006). An application of the Dempster-Shafer theory of evidence to the classification of knee function and detection of improvement due to total knee replacement surgery. *Journal of Biomechanics*.

[19] Takahashi T., Ishida K., Hirose D. (2004). Vertical ground reaction force shape is associated with gait parameters, timed up and go, and functional reach in elderly females. *Journal of Rehabilitation Medicine*.

[20] Zhu W. X., Zhou J. J., Wu Y. (2014). Gait abnormality detected of pigs based on machine vision. *Applied Mechanics and Materials*.

[21] Pham T. T., Moore S. T, Lewis S. J. G (2017). Freezing of gait detection in Parkinson’s disease: a subject-independent detector using anomaly scores. *IEEE Transactions on Bio-Medical Engineering*.

[22] Figueiredo J., Santos C. P., Moreno J. C. (2018). Automatic recognition of gait patterns in human motor disorders using machine learning: a review. *Medical Engineering & Physics*.

[23] Markou M., Singh S. (2003). Novelty detection: a review-part 2:. *Signal Processing*.

[24] Wong W.-K., Moore A. W., Cooper G. F., Wagner M. M. Bayesian network anomaly pattern detection for disease outbreaks.

[25] Rousseeuw P. J., Driessen K. V. (1999). A fast algorithm for the minimum covariance determinant estimator. *Technometrics*.

[26] Tax D. M. J., Duin R. P. W. (2004). Support vector data description. *Machine Learning*.

[27] Breunig M. M., Kriegel H.-P., Ng R. T., Sander J. LOF: identifying density-based local outliers.

[28] Liu F. T., Ting K. M., Zhou Z.-H. Isolation forest.

[29] He Z., Xu X., Deng S. (2003). Discovering cluster-based local outliers. *Pattern Recognition Letters*.

[30] Hanson M. A., Powell H. C., Barth A. T., Lach J., Brandt-Pearce M. Neural network gait classification for on-body inertial sensors.

[31] Parkka J., Ermes M., Korpipaa P., Mantyjarvi J., Peltola J., Korhonen I. (2006). Activity classification using realistic data from wearable sensors. *IEEE Transactions on Information Technology in Biomedicine*.

[32] Lai D. T. H., Levinger P., Begg R. K., Gilleard W. L., Palaniswami M. (2009). Automatic recognition of gait patterns exhibiting patellofemoral pain syndrome using a support vector machine approach. *IEEE Transactions on Information Technology in Biomedicine*.

[33] Joshi D., Khajuria A., Joshi P. (2017). An automatic non-invasive method for Parkinson’s disease classification. *Computer Methods and Programs in Biomedicine*.

[34] Elkholy A., Makihara Y., Gomaa W., Ahad M. A. R., Yagi Y. Unsupervised GEI-based gait disorders detection from different views.

[35] Yang K., Ahn C. R., Vuran M. C., Aria S. S. (2016). Semi-supervised near-miss fall detection for ironworkers with a wearable inertial measurement unit. *Automation in Construction*.

[36] Mikos V. Real-time patient adaptivity for freezing of gait classification through semi-supervised neural networks.

[37] Li Y., Yin Y., Liu L., Pang S., Yu Q. Semi-supervised gait recognition based on self-training.

[38] Han J., Bhanu B. (2005). Individual recognition using gait energy image. *IEEE Transactions on Pattern Analysis and Machine Intelligence*.

[39] Liu J., Zheng N. Gait history image: a novel temporal template for gait recognition.

[40] Ma Q., Wang S., Nie D., Qiu J. Recognizing humans based on gait moment image.

[41] Chen C., Liang J., Zhao H., Hu H., Tian J. (2009). Frame difference energy image for gait recognition with incomplete silhouettes. *Pattern Recognition Letters*.

[42] Yao L., Kusakunniran W., Wu Q., Zhang J., Tang Z., Yang W. (2019). Robust gait recognition using hybrid descriptors based on Skeleton Gait Energy Image. *Pattern Recognition Letters*.

[43] Yu S.-S., Chu S.-W., Wang C.-M., Chan Y.-K., Chang T.-C. (2018). Two improved k-means algorithms. *Applied Soft Computing*.

[44] Wagstaff K., Cardie C., Rogers S., Schrödl S. Constrained k-means clustering with background knowledge.

[45] Basu S., Banerjee A., Mooney R. J. Active semi-supervision for pairwise constrained clustering.

[46] Zhigang C., Xuan L., Fan Y. Constrained k-means with external information.

[47] He Z. (2016). Evolutionary *K*-Means with pair-wise constraints. *Soft Computing*.

[48] Yang Z., Wu A. (2020). A non-revisiting quantum-behaved particle swarm optimization based multilevel thresholding for image segmentation. *Neural Computing and Applications*.

[49] Bolón-Canedo V., Alonso-Betanzos A. (2019). Ensembles for feature selection: a review and future trends. *Information Fusion*.

[50] Yang K., Ahn C. R., Vuran M. C., Kim H. Sensing workers gait abnormality for safety hazard identification.

[51] Wang L., Tan T., Hu W., Ning H. (2003). Automatic gait recognition based on statistical shape analysis. *IEEE Transactions on Image Processing: A Publication of the IEEE Signal Processing Society*.

